# Identification of novel candidate loci and genes for seed vigor-related traits in upland cotton (*Gossypium hirsutum* L.) via GWAS

**DOI:** 10.3389/fpls.2023.1254365

**Published:** 2023-09-01

**Authors:** Libei Li, Yu Hu, Yongbo Wang, Shuqi Zhao, Yijin You, Ruijie Liu, Jiayi Wang, Mengyuan Yan, Fengli Zhao, Juan Huang, Shuxun Yu, Zhen Feng

**Affiliations:** ^1^ The Key Laboratory for Quality Improvement of Agricultural Products of Zhejiang Province, College of Advanced Agricultural Sciences, Zhejiang A&F University, Lin’an, Hangzhou, China; ^2^ Cotton Sciences Research Institute of Hunan, Changde, Hunan, China; ^3^ Cotton and Wheat Research Institute, Huanggang Academy of Agricultural Sciences, Huanggang, Hubei, China; ^4^ State Key Laboratory of Rice Biology and Breeding, China National Rice Research Institute, Hangzhou, China; ^5^ Research Center of Buckwheat Industry Technology, Guizhou Normal University, Guiyang, China

**Keywords:** upland cotton, seed vigor, germination rate, GWAS, candidate genes

## Abstract

Seed vigor (SV) is a crucial trait determining the quality of crop seeds. Currently, over 80% of China’s cotton-planting area is in Xinjiang Province, where a fully mechanized planting model is adopted, accounting for more than 90% of the total fiber production. Therefore, identifying SV-related loci and genes is crucial for improving cotton yield in Xinjiang. In this study, three seed vigor-related traits, including germination potential, germination rate, and germination index, were investigated across three environments in a panel of 355 diverse accessions based on 2,261,854 high-quality single-nucleotide polymorphisms (SNPs). A total of 26 significant SNPs were detected and divided into six quantitative trait locus regions, including 121 predicted candidate genes. By combining gene expression, gene annotation, and haplotype analysis, two novel candidate genes (*Ghir_A09G002730* and *Ghir_D03G009280*) within *qGR-A09-1* and *qGI/GP/GR-D03-3* were associated with vigor-related traits, and *Ghir_A09G002730* was found to be involved in artificial selection during cotton breeding by population genetic analysis. Thus, understanding the genetic mechanisms underlying seed vigor-related traits in cotton could help increase the efficiency of direct seeding by molecular marker-assisted selection breeding.

## Introduction

Upland cotton (*Gossypium hirsutum* L.) is one of the world’s most important cash crops and a major source of natural fibers, accounting for more than 95% of global cotton production (Chen et al., 2007). Lint yield depends largely on the quality of cotton seeds, while seed vigor (SV) is crucial for evaluating seed quality (Sawan, 2016). SV also determines the growth of crops and food safety; for example, rapidly and uniformly germinating seeds can significantly increase the emergence rate in the field and suppress weed growth (He et al., 2019a). In addition, with the widespread application of mechanized direct seeding (DS) in cotton production, cotton seeds with low vigor will make it difficult to sow all seedlings at once, leading to many problems such as uneven seedling age and weak seedling vigor (Qun et al., 2007; Liu et al., 2015). Therefore, the identification of loci and genes related to SV is urgently needed for DS of cotton.

Seed germination is a key factor affecting SV traits in plants. Phytohormones such as gibberellin (GA) and abscisic acid (ABA) have been reported to be essential for the regulation of seed germination (Yamaguchi, 2008; Ryu and Cho, 2015)—for example, GA and ABA synthesis pathway-related genes (*GA20ox3, GA3ox1*, *GA2ox5*, *ABI3*, and *ABI5*) have a strong effect on seed germination (Yamauchi et al., 2004; Yamaguchi, 2008; Iglesias-Fernandez and Matilla, 2009). When plants are under abiotic stress, ABA in the plant will increase rapidly, and high levels of ABA will close the stomata and activate complex signaling pathways mediated by kinase/phosphatase regulation (Kim et al., 2010). Low levels of reactive oxygen species (ROS) act as signaling particles to promote dormancy release and trigger seed germination (Li et al., 2022)—for example, *OsCDP3.10* promotes the accumulation of H_2_O_2_ during the early stage of seed germination by increasing the amino acid content (Peng et al., 2022). The relationship between seed germination and the ROS scavenging system has been validated in many crops and other plants, such as *Arabidopsis* (Leymarie et al., 2012), wheat (Ishibashi et al., 2008), and rice (Ye et al., 2012). Furthermore, crosstalks between ABA and ROS signaling pathways have also been reported in plants. In rice, *qSE3* significantly increased ABA biosynthesis and activated ABA signaling responses, resulting in decreased H_2_O_2_ levels in germinating seeds under salinity stress (He et al., 2019b).

SV-related traits are quantitative traits controlled by both genetic and environmental factors (Li W. et al., 2021). These traits include germination rate (GR), germination percentage (GP), germination index (GI), vigor index (VI), seedling shoot length (SL), and shoot fresh weight (FW) (Dai et al., 2022; Si et al., 2022). In recent years, linkage mapping has been widely used to identify SV-related quantitative trait loci (QTLs) in crops, and multiple QTLs have been cloned (Fujino et al., 2004; Fujino et al., 2008; He et al., 2019b; Jiang et al., 2020; Veisi et al., 2022). By using BC_1_F_5_ populations derived from a rice intraspecific cross (‘WTR-1’ × ‘Y134’), 28 SV-related QTLs were identified by a SNP genotyping array, and one major QTL (*q1stGC_11.2_*) explaining 19.9% of the phenotypic variation (PV) was flanked by SNP_11_27994133 on chromosome 11 (Dimaano et al., 2020). In wheat, a total of 49 QTLs were detected on 12 chromosomes, including seven SV candidate genes involved in the processes of cell division during germination of aged seeds, carbohydrate and lipid metabolism, and transcription (Shi et al., 2020). Wang L. et al. (2022) constructed a linkage map based on specific-locus-amplified fragment sequencing (SLAF-seq) SNP markers in melon; *2020/2021-qsg5.1* was significant in both environments, and *MELO3C031219.2*, in this region, exhibited a significant expression difference between the parental lines during multiple germination stages (Wang L. et al., 2022). Under low temperature conditions, three QTLs (*qLTG-3-1*, *qLTG3-2*, and *qLTG-4*) related to GR were identified by 122 backcross inbred lines, and the phenotypic variation explained (PVE) by *qLTG-3-1* was 35.0% (Fujino et al., 2004). Subsequently, *qLTG-3-1* was cloned, which was closely related to tissue vacuolation, by covering the embryo (Fujino et al., 2008). Furthermore, the genome-wide association study (GWAS) approach is a method in which germplasm resources are used to study the genetic structure of target traits. Compared to traditional QTL mapping, GWAS can provide higher resolution by using ancestral recombination events and has been successfully applied to identify significant SNP loci and potential candidate genes associated with important agronomic traits in major crops (Zhu et al., 2008; Shikha et al., 2021)—for example, SV-related QTLs were identified in 346 rice accessions using GWAS, while 51 significant SNPs were detected for SL, GR, and FW (Dai et al., 2022). In addition, a previous study involving 187 rice accessions identified the candidate gene *OsSAP16*; the loss of *OsSAP16* function reduced the rice seed germination rate (Wang et al., 2018). Recently, a candidate gene (*Gh_A09G1509*) responsible for seed germination was detected through a GWAS panel in upland cotton by using whole-genome resequencing (Si et al., 2022). These results suggest that genome-wide association analysis is an effective method for identifying genes associated with seed germination.

To date, many quantitative traits have been reported in cotton, such as fiber quality traits (Su et al., 2016b; Zhang et al., 2019), early maturity traits (Li et al., 2017; Li L. et al., 2021), and yield component traits (Su et al., 2016a; Feng et al., 2022). However, SV-related traits in cotton have received little attention, and most research have focused on seed germination in relation to stress tolerance (Yuan et al., 2019; Chen L. et al., 2020; Gu et al., 2021; Guo et al., 2022). Few candidate genes for cotton SV-related traits have been identified (Si et al., 2022), and the mechanism of seed germination needs further study. In this study, GR, GP, and GI were determined in a natural population of upland cotton in three environments, and whole-genome resequencing was used to achieve deep coverage and obtain high-quality SNP markers. In addition, six stable QTLs and two novel candidate genes (*Ghir_A09G002730* and *Ghir_D03G009280*) for SV-related traits were further identified by a GWAS panel, laying the foundation for understanding the genetic mechanism underlying SV and providing potential information for applying these potential elite loci for marker-assisted selection (MAS) in cotton breeding.

## Materials and methods

### GWAS population and field experiments

The 355 upland cotton germplasm resources collected by laboratories worldwide represent a natural population. Previous studies focused on early maturity (Li L. et al., 2021), fiber quality (Su et al., 2016b), fiber yield (Su et al., 2016a; Feng et al., 2022), and plant architecture component traits based on abundant phenotypic variations in this population (Su et al., 2018). These upland cotton varieties are from different countries and represent accessions resulting from more than 100 years of global upland cotton breeding. Seeds of the GWAS population used for phenotyping SV-related traits were collected from three environments, including Huanggang in Hubei Province (30°57′ N, 114°92′ E) in 2021 (E1: Huanggang-2021) and Sanya in Hainan Province (18°36′ N, 109°17′ E) in two consecutive years (2021 and 2022) (E2: Sanya-2021 and E3: Sanya-2022). The field experiments in Sanya and Huanggang were conducted following a randomized complete block design with two and three replications, respectively.

### Phenotyping for SV-related traits and statistical analysis

The phenotyping of SV-related traits was carried out by the sandponic method based on previously described methods (Si et al., 2022). Cotton seeds collected from the field were ginned, and cotton fuzz was removed by concentrated sulfuric acid. Then, all seeds were sun-dried for 2 days to break dormancy uniformly. A total of 150 plump seeds with uniform size and full grain were selected, disinfected with 15% sodium hypochlorite for 10 min, and then washed clean with distilled water. Then, each line was evenly planted in a plastic sand box containing 800 g of dry quartz sand with a size of 13 cm × 19 cm × 12 cm. Subsequently, the seeds were covered with 250 g of dry quartz sand, and 200 mL of distilled water was added. The number of germinated seeds was counted each day until the seventh day. All experiments were conducted in a phytotron with 16 h of light (25°C) and 8 h of darkness (18°C). Three biological replicates were included for each accession, and 50 seeds were used for each replicate. Moreover, three SV-related traits (GR, GP, and GI) were selected for measurement. The full name, abbreviation, and measurement method of each trait are listed in **Table 1** as described by Yuan et al. (2019). The statistical analysis of the maximum value, minimum value, average value, etc., was performed using R software (version: 4.2.2).

**Table 1:** Method of measurement for seed vigor-related traits.

Trait	Trait abbreviation	Measurement methods for each trait
Germination potential	GP	The number of germinated seeds in the early stage of germination (3 days)/the number of seeds tested
Germination rate	GR	The number of germinated seeds on the 7th day after planting/the number of tested seeds
Germination index	GI	GI = ∑(Gt/Dt), where Gt represents the number of germinated seeds per day and Dt represents the number of days corresponding to Gt

### Development of SNP markers

The resequencing data (PRJNA389777) of the 355 upland cotton germplasms used in this study were reported in a previous study (Li L. et al., 2021). The Illumina HiSeq4000 platform was used for paired-end read sequencing, with an average sequencing depth of more than 10×. Based on previously released data, the new variation map of the natural population was employed in the ‘HaplotypeCaller’ module of GATK (version: 4.2.6.1) (Mckenna et al., 2010). Briefly, the variation detection process was as follows: (1) The quality of paired-end reads from 355 accessions was assessed using FastQC (version: 0.11.9) (Andrews, 2010); (2) Sequencing quality control was carried out with fastp software (version: 0.23.2) to obtain high-quality reads with the following parameters: ‘-w 16 -c -l 80 -5 -3 -W 4 -M 20 -f 10 -F 13 -t 3 -T 3 -q 20 -u 40’ (Chen et al., 2018); (3) All high-quality reads were mapped to the ‘TM-1’ (version: HAU_v1.1) reference genome using BWA (version: 0.7.17-r1188) (Li, 2013; Wang et al., 2019); (4) Then, Picard software (https://github.com/broadinstitute/picard) was used to sort the BAM file and mark duplicate reads; (5) The ‘HaplotypeCaller’ module of GATK (version: 4.2.6.1) was used to identify variant sites and perform SNP filtering with the following conditions: ‘QUAL <30, DP <1,340, DP >10,050, QD <2.0, MQ <35, FS >70, SOR >3, MQRankSum <-12.5, and ReadPosRankSum <-4.0’; (6) The SNP clusters with at least three SNPs detected within a 10-base window were removed; (7) SNPs within five base pairs of an InDel were filtered out by BCFtools software (version: 0.1.19-44428cd) (Danecek et al., 2021); and (8) SNPs with a minor allele frequency (MAF) <5% and missing rate <20% were discarded by VCFtools (version: 0.1.16) (Danecek et al., 2011).

### GWAS and genetic diversity analysis

Genome-wide association analysis was performed by combining 2,262,367 high-quality SNPs with the phenotype data of 355 upland cotton accessions collected in three environments for SV-related traits using linear mixed models in GEMMA (version: 0.98.3) and executed by vcf2gwas software (version: 0.8.7) (Zhou and Stephens, 2012; Vogt et al., 2022). *P <*1 × 10^-6^ was used as the threshold to detect significant SNP loci. Additionally, the PVE by each marker was calculated as previously reported (Feng et al., 2022). The nucleotide diversity (*π*)a value was calculated using VCFtools based on the release years (before the 1950s, 1950s–1970s, 1980s–1990s, and 2000s–2020s) and geographical distribution (early maturity region: NSER, Yellow River region: YRR, Yangtze River region: YZRR, and Northwest Inland region: NIR) of the 355 accessions. The packages ‘CMplot’ (https://github.com/YinLiLin/CMplot), ‘LDheatmap’ (Shin et al., 2006), and ‘ggplot2’ (Wickham, 2011) in R software were used to generate Manhattan plots and for linkage disequilibrium (LD) block analysis and haplotype analysis.

### Candidate gene identification and expression analysis

Based on the ‘TM-1’ reference genome (HAU_v1.1) (Wang et al., 2019), the genes in the interval located 200 kb upstream and downstream of the significant SNPs were defined as candidate genes. The protein sequences of the candidate genes were obtained from Cottongene (https://www.cottongen.org/). Then, local BLAST software was used to compare the protein sequence of the candidate gene with the *Arabidopsis* protein database (https://www.arabidopsis.org) to obtain the homologous sequence, and the criterion was set to less than E^-60^ (Johnson et al., 2008). The expression patterns of SV candidate genes in upland cotton were determined by RNA-seq and quantitative reverse-transcription PCR (qRT-PCR) analysis. RNA isolation method was performed as described by Feng et al. (2022). *GhUBQ7* was used as an internal control. Quantitative analysis method was performed using a Roche real-time qPCR system (Light Cycler 480 II) and SYBR with three biological repeats. The public RNA-seq data (PRJNA248163) including SRR1695160, SRR1695161, and SRR1695162 were downloaded from NCBI (https://www.ncbi.nlm.nih.gov/bioproject/). The Illumina Hiseq2000 platform was used to perform RNA sequencing on ‘TM1’ seeds soaked in water for 0, 5, and 10 h, and the paired-end clean reads length was more than 100 bp. The gene expression values were normalized by the average expression levels (log2) based on transcripts per million values. The clustered heat map was drawn by the R package ‘pheatmap’ (Kolde, 2012).

## Results

### Characterization and distribution of SNPs in the upland cotton genome

**Figure 1:**
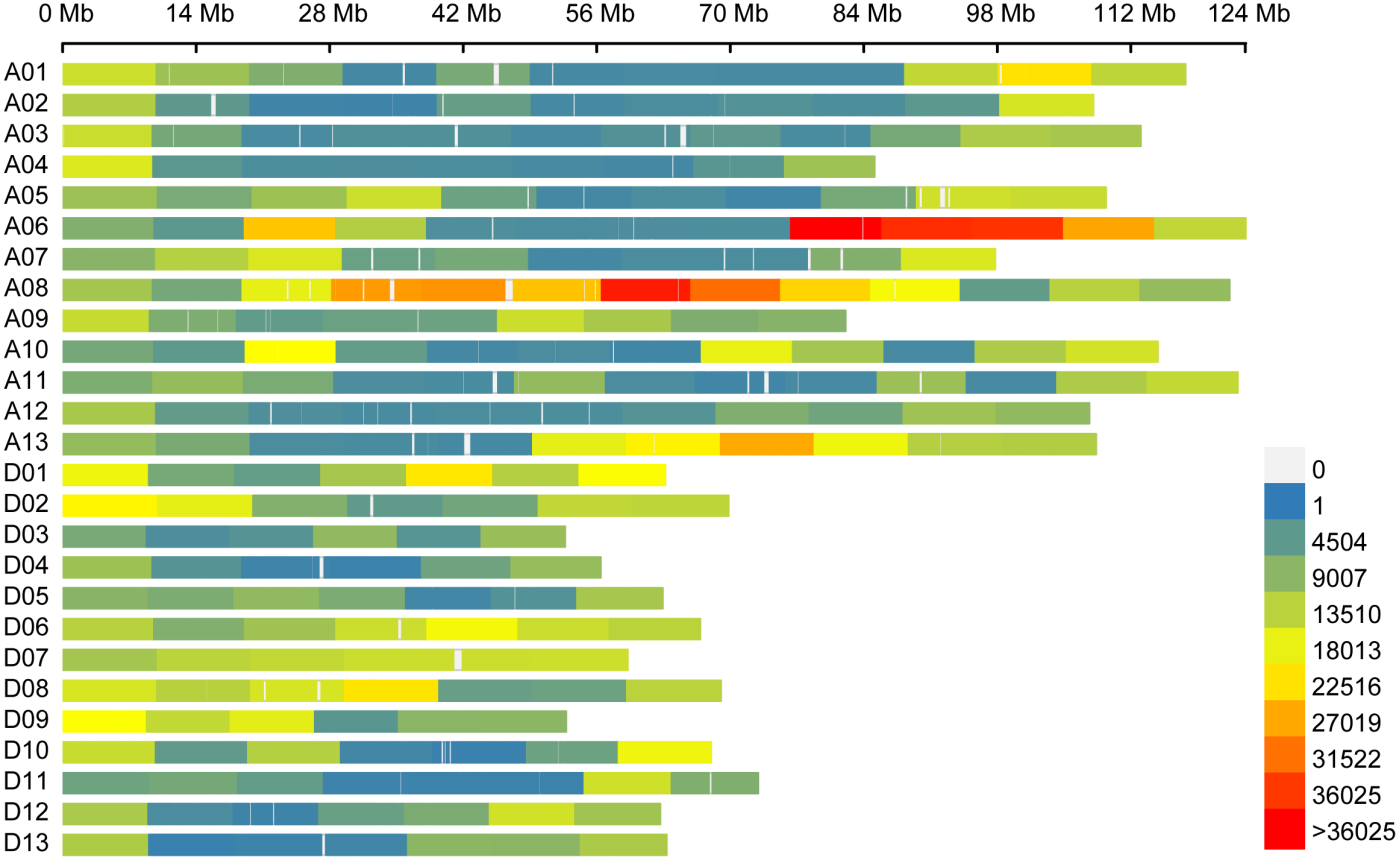
Single-nucleotide polymorphism distributions in the upland cotton genome. The number of SNPs within a 10-Mb window. A01–A13 and D01–D13 on the Y axis are the numbers of the 26 chromosomes. The X axis represents chromosome length (Mb).

Resequencing of the natural population libraries by the Illumina HiSeq 4000 platform with 150 bp paired-end reads, as described in previous reports (Li L. et al., 2021), yielded approximately 65,013 million reads in total for the 355 cotton genotypes. Approximately 88.3% of the total bases were successfully mapped to the cotton reference genome, and the statistical sequencing depth corresponded to 11.7-fold in the 355 upland cotton accessions. A total of 2,262,367 SNPs distributed across the cotton genome with a MAF >0.05, and missing rate of resequencing data of less than 20% was used for the GWAS of the 355 cotton germplasm accessions, of which the At and Dt subgenomes contained 1,404,637 and 857,730 SNPs, resulting in an average SNP density of 993.44 and 1045.91 SNP/Mb, respectively (**Table 2**; **Figure 1**). The percentage of the SNPs in each chromosome varied from 1.4% on chromosome D04 to 11.4% on chromosome A08 (**Figure 1**). Most of the SNPs were located in intergenic regions (84.38%), whereas the exonic and intronic genome regions contained only 0.89% and 3.03% of SNPs, respectively (**Supplementary Table S1**). In addition, SNPs in the coding regions (coding sequences, CDSs) included 33.26% synonymous mutations and 64.13% nonsynonymous mutations.

**Table 2:** Distribution and frequency of single-nucleotide polymorphisms (SNPs) identified using the resequencing approach in upland cotton.

Chromosome	Chromosome length (Mb)	SNP number	Density (SNP/Mb)	Chromosome	Chromosome length (Mb)	SNP number	Density (SNP/Mb)
A01	117.76	102,597	871.25	D01	63.21	97,337	1,539.92
A02	108.09	56,850	525.94	D02	69.84	86,010	1,231.56
A03	113.06	73,858	653.27	D03	52.70	37,138	704.70
A04	85.15	48,890	574.16	D04	56.43	33,068	586.00
A05	109.42	93,469	854.23	D05	62.93	49,985	794.25
A06	124.06	216,693	1,746.73	D06	66.87	95,435	1,427.18
A07	97.78	82,817	846.95	D07	59.26	85,111	1,436.29
A08	122.38	259,187	2,117.94	D08	69.04	93,091	1,348.38
A09	82.10	82,034	999.16	D09	52.82	74,192	1,404.64
A10	114.85	102,498	892.44	D10	68.01	59,948	881.51
A11	123.21	85,696	695.52	D11	72.94	44,642	612.02
A12	107.67	65,645	609.67	D12	62.69	55,606	886.94
A13	108.38	134,403	1,240.15	D13	63.34	46,167	728.84
Total	1,413.91	1,404,637	993.44	Total	820.08	857,730	1,045.91

### PV of SV-related traits

**Figure 2:**
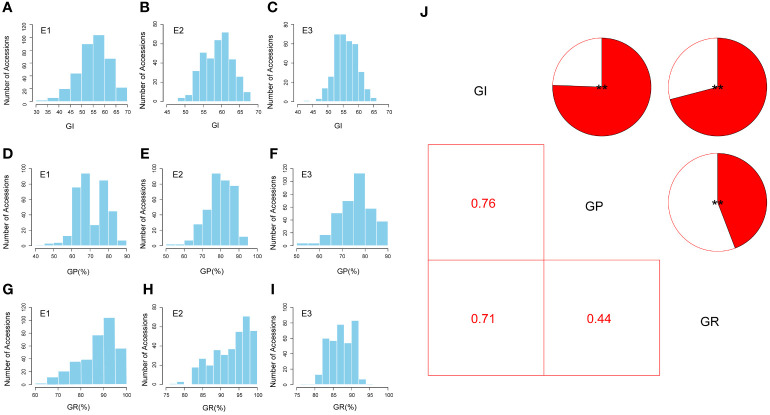
Phenotypic variation analysis of seed vigor (SV)-related traits. **(A–I)** Distributions of the mean values for the germination index (GI), germination potential (GP), and germination rate (GR) in three environments, respectively. **(J)** Correlation analysis of SV-related traits (GI, GP, and GR) in three environments (**P < 0.01). E1, Huanggang-2021; E2, Sanya-2021; and E3, Sanya-2022.

The three SV-related traits (GI, GP, and GR) of natural population accessions were measured in three environments. The values followed a normal distribution for the GI and GP but showed a skewed distribution for GR based on Shapiro–Wilk tests (**Supplementary Table S2**). The frequency histograms of SV-related traits are shown in **Figures 2A–I**. The lowest average GI was 55.23 in the E1 environment, and the highest average GI was 58.92 in the E2 environment, with a coefficient of variation (CV) ranging from 6.52% to 12.02% (**Supplementary Table S2**). For GP, the E1 environment had the lowest average value of 70.92%, while the E2 environment had the highest average value of 79.61%; the CV in the E1 environment (11.67%) was higher than that in the E2 environment (9.21%) and the E3 environment (9.37%) (**Supplementary Table S2**). For GR, the lowest average value was 87.37% in the E1 environment, and the highest average value was 93.25% in the E2 environment, with a CV ranging from 3.71% to 10.48% (**Supplementary Table S2**). Two-way analysis of variance (ANOVA) showed that genotype (G) and the genotype-by-environment interaction (G × E) had significant effects on the GI, GP, and GR (*P* < 0.001) (**Supplementary Table S3**). Furthermore, the heritability of these three SV-related traits ranged from 74.23% (GR) to 81.75% (GP), whereas that of GI was 76.03% (**Supplementary Table S3**). These results suggested that SV-related traits have extensive PV in the GWAS panel, which is suitable for further GWAS.

### GWAS of SV-related Traits in Upland Cotton

**Figure 3:**
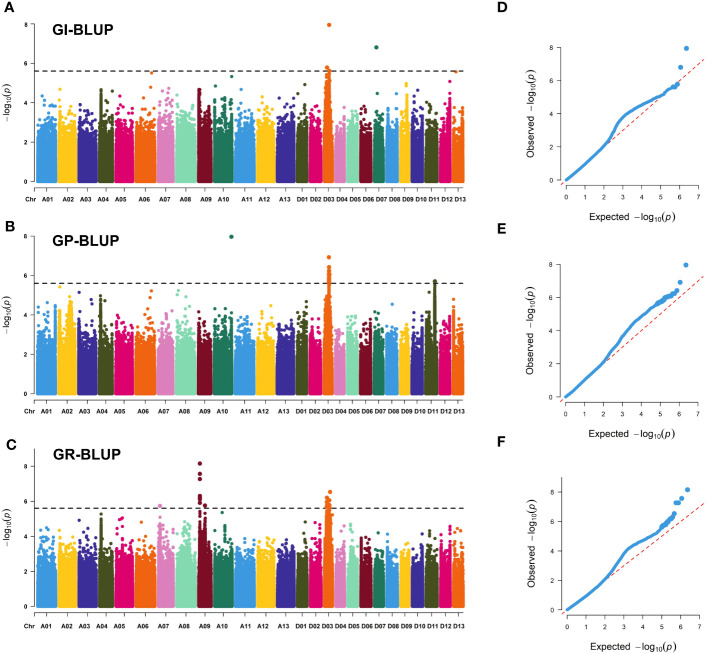
Genome-wide association study results for seed vigor-related traits. **(A–C)** Manhattan plots of GI-BLUP, GP-BLUP, and GR-BLUP for single-nucleotide polymorphism (SNP) markers, respectively. Significant SNP markers are distinguished by black lines. **(D–F)** QQ plots for GI-BLUP, GP-BLUP, and GR-BLUP, respectively.

A total of 292 significant SNPs for three SV-related traits were identified on 11 chromosomes using the linear mixed model (**Figure 3**; **Supplementary Table S4**; **Supplementary Figures S1**-**S3**). Only 11 SNPs were identified in the At subgenome, whereas 281 SNPs were localized to the Dt subgenome. Among them, chromosome D03 had the highest number of SNPs (281), with a total of 254, and the range of -log_10_(*p*) values was from 6.00 to 8.27. Furthermore, 26 stable SNPs were identified in a minimum of two environments (including for the best linear unbiased predictor, BLUP) or two traits, which were declared as six stable QTLs, focusing on chromosomes A09, A10, and D03. Notably, a QTL region (*qGR-A09-1*) located on chromosome A09 showed a strong SNP cluster associated with GR, which had a PVE of 6.76–8.56% and -log_10_(*P*) ranging from 6.19 to 7.74. *qGP-A10-1* on chromosome A10 had only one SNP that explained 8.15% of the observed PVE, with a LOD score of 7.39. Four QTLs on chromosome D03 (*qGR/GI-D03-1*, *qGI/GR-D03-2*, *qGI/GP/GR-D03-3*, and *qGI/GP/GR-D03-4*) were identified in two, three, three, and four environments, explaining 6.61–7.39%, 6.72–7.79%, 6.65–8.43%, and 6.61–8.90% of the observed PVE, respectively. Interestingly, a stable QTL (*qGI/GP/GR-D03-3*) region was revealed on chromosome D03 from 31.68 to 32.61 Mb and was flanked by regions associated with the GI, GP, and GR in the E1, E3, and BLUP environments. Thus, the QTLs *qGR-A09-1* and *qGI/GP/GR-D03-3* could be treated as major QTLs for further dissection.

### Identification of a candidate gene for GR on chromosome A09

**Figure 4:**
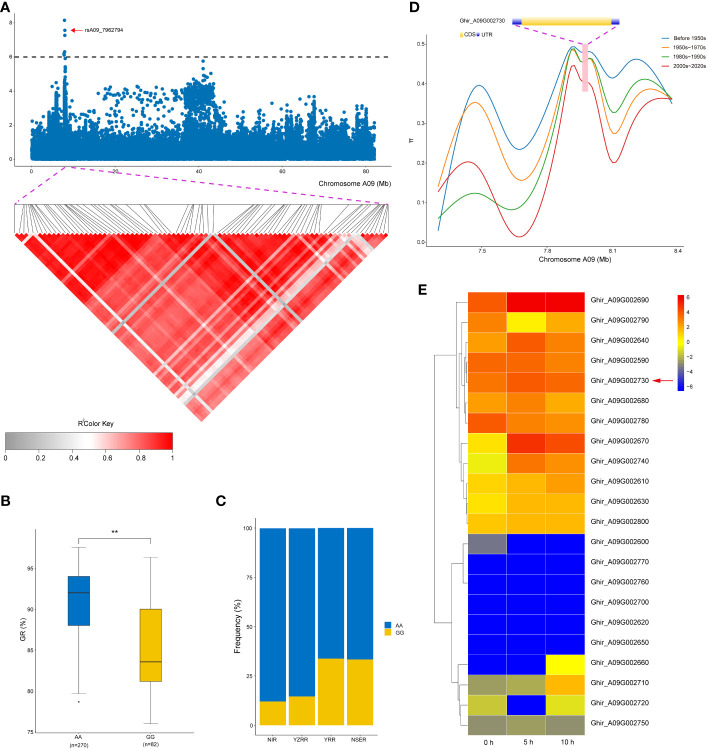
Variation analysis of the germination rate (GR)-associated gene Ghir_A09G002730 in the candidate region. **(A)** Local Manhattan plots for GR-related genes on chromosome A09 and linkage disequilibrium heat map for the candidate region within 21.9 kb. **(B)** Box plots for GR of the two haplotypes mentioned above (**P < 0.01). **(C)** Differentiation of the genetic diversity distribution of *Ghir_A09G002730* in four geographic areas (NIR, Northwest Inland region; YZRR, Yangtze River region; YRR, Yellow River region; and NSER, Northern Specific Early-Maturity region). **(D)** Gene structure diversity of *Ghir_A09G002730* across three breeding stages. **(E)** Heat map of candidate gene expression patterns in the seed germination stage (0, 5, and 10 h) on chromosome A09.

In this study, a novel QTL, *qGR-A09-1*, exhibited a significant SNP cluster (rsA09_7745467, rsA09_7791621, rsA09_7878527, rsA09_7908017, rsA09_7954329, rsA09_7954353, and rsA09_7962794) occupying a physical region of 0.2 Mb on chromosome A09 (**Figure 4A**). Meanwhile, 22 genes were annotated in this QTL region based on the *G*. *hirsutum* reference genome (Wang et al., 2019), except for *Ghir_A09G002720* and *Ghir_A09G002760*, which did not have annotation information (**Supplementary Table S5**). We further conducted LD analysis on the significant SNP rsA09_7962794, and LD blocks were found in this region (**Figure 4A**). In this QTL interval, rsA09_7962794 on chromosome A09 showed a strong association with GR, with 7.95% of the PVE downstream of *Ghir_A09G002730* (**Table 3**). rsA09_7962794 had two haplotypes, GG and AA, which resulted in the accessions carrying the AA genotype having a significantly higher GR than those carrying the GG haplotype in three environments (*P* < 0.01) (**Figure 4B**). In addition, to gain a further understanding of the genetic characteristics of rsA09_7962794 in relation to geographic distribution, the 355 upland cotton accessions were divided into four groups: NIR, YZRR, YRR, and NSER. Interestingly, YRR and NSER showed an extraordinarily low frequency of the nonfavorable haplotype (GG), while the accessions obtained from YZRR and NIR had a relatively high frequency of the favorable haplotype (AA) (>75%) (**Figure 4C**). Furthermore, the genetic diversity of *Ghir_A09G002730* decreased following the breeding period. Cotton accessions released before the 1980s showed greater diversity than accessions bred from the 1980s to the 2000s, while accessions bred after the 2000s showed the lowest diversity (**Figure 4D**). Specifically, *Ghir_A09G002730* belongs to the pentatricopeptide repeat (PPR) superfamily protein family and has higher expression levels during the seed germination stage from 0 to 10 h than other genes (**Figure 4E**). The qRT-PCR analysis also showed that *Ghir_A09G002730* had higher expression levels in the accessions (‘Liaomian27’ and ‘Xinluzhong35’) carrying the AA allele than in accessions (‘PB12-1-8’ and ‘Xiazao2’) with GG allele during the seed germination stage (**Supplementary Figure S4**).

**Table 3:** Significant quantitative trait locus (QTLs) associated with seed vigor-related traits.

QTLs	SNP	Chromosome	Position (bp)	Trait	Environment	Allele	-log_10_(*P*)	Phenotypic variation explained (%)
qGR-A09-1	rsA09_7745467	A09	7,745,467	GR	BLUP; E1	T/C	6.19	6.76
	rsA09_7791621		7,791,621	GR	BLUP; E1	A/G	6.25	6.82
	rsA09_7878527		7,878,527	GR	BLUP; E1; E2	T/C	7.74	8.56
	rsA09_7908017		7,908,017	GR	BLUP; E1	A/G	6.20	6.76
	rsA09_7954329		7,954,329	GR	BLUP; E1; E2	G/C	6.88	7.55
	rsA09_7954353		7,954,353	GR	BLUP; E1; E2	A/G	6.88	7.55
	rsA09_7962794		7,962,794	GR	BLUP; E1; E2	G/A	7.22	7.95
qGP-A10-1	rsA10_112752002	A10	112,752,002	GP	BLUP; E2; E3	C/T	7.39	8.15
qGR/GI-D03-1	rsD03_15149331	D03	15,149,331	GI	E1	C/T	6.74	7.39
				GR	E1; E2	C/T	6.07	6.61
	rsD03_15180622	D03	15,180,622	GI	E1	T/C	6.08	6.62
				GR	E2	T/C	6.24	6.81
qGI/GR-D03-2	rsD03_16442805	D03	16,442,805	GR	BLUP; E2	T/A	6.25	6.81
	rsD03_17044820	D03	17,044,820	GI	E1	A/G	6.16	6.72
				GR	E2	A/G	6.40	6.99
	rsD03_17639861	D03	17,639,861	GI	E1	A/T	7.08	7.79
				GR	E2	A/T	6.49	7.10
qGI/GP/GR-D03-3	rsD03_31686969	D03	31,686,969	GP	BLUP; E1	A/C	6.61	7.24
	rsD03_31912853	D03	31,912,853	GI	BLUP; E1	C/T	7.64	8.43
				GP	BLUP; E1	C/T	7.40	8.16
	rsD03_32121851	_D03	32,121,851	GP	BLUP; E1	G/A	6.85	7.52
	rsD03_32123311	D03	32,123,311	GP	E1	A/G	7.12	7.84
				GR	E3	A/G	6.39	6.98
	rsD03_32217200	D03	32,217,200	GP	BLUP; E1	A/G	7.03	7.72
	rsD03_32235852	D03	32,235,852	GP	E1	T/C	6.83	7.49
				GR	E3	T/C	6.10	6.65
	rsD03_32407516	D03	32,407,516	GP	BLUP; E1	A/G	6.64	7.28
	rsD03_32411896	D03	32,411,896	GP	BLUP; E1	T/C	7.04	7.74
	rsD03_32414028	D03	32,414,028	GP	BLUP; E1	G/A	6.67	7.31
	rsD03_32429655	D03	32,429,655	GP	BLUP; E1	G/A	7.31	8.05
	rsD03_32518414	D03	32,518,414	GP	BLUP; E1	A/G	7.12	7.83
	rsD03_32611645	D03	32,611,645	GP	BLUP; E1	A/G	6.86	7.53
qGI/GP/GR-D03-4	rsD03_36696073	D03	36,696,073	GI	E1	A/C	6.07	6.61
				GP	E1	A/C	8.04	8.90
				GR	BLUP; E2; E3	A/C	6.53	7.15

### Identification of a candidate gene for GR on chromosome D03

**Figure 5:**
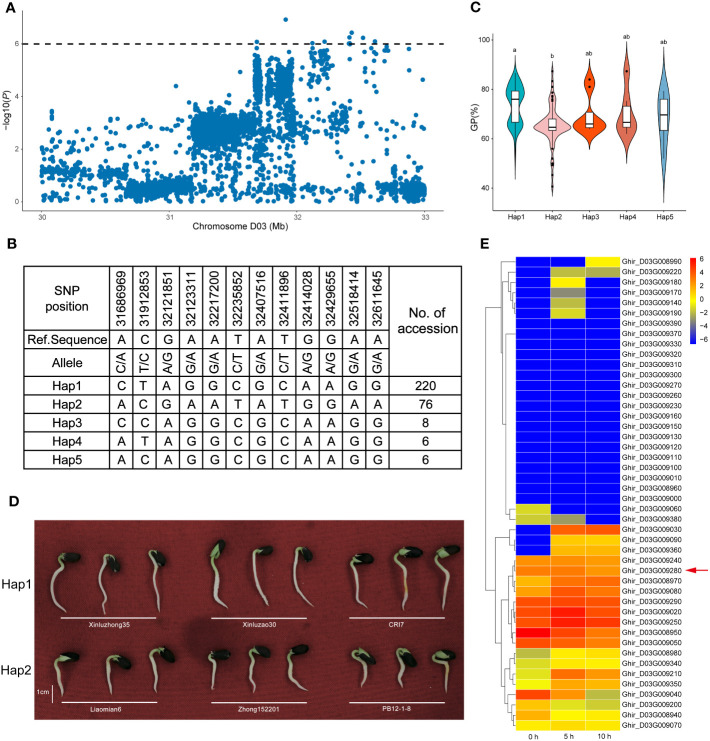
Variation analysis of seed vigor (SV)-related traits associated with qGI/GP/GR-D03-3 in the candidate region. **(A)** Local Manhattan plots for SV-related genes on chromosome D03 from 30 to 33 Mb. **(B)** Top two haplotypes of *qGI/GP/GR-D03-3* in 355 upland cotton accessions. **(C)** Comparison of germination potential between accessions containing Hap1, Hap2, Hap3, Hap4, and Hap5. Letters on the violin plot indicate significant differences according to one-way ANOVA (LSD test; P < 0.05). **(D)** Comparison of seed germination status for 3 days between Hap1 and Hap2. **(E)** Heat map of candidate gene expression patterns in the seed germination stage (0, 5, and 10 h) on chromosome D03.

As mentioned above, another distinct SNP enrichment QTL region, *qGI/GP/GR-D03-3*, was detected for the GI, GP, and GR across multiple environments, which could explain the relatively high PVE of 6.65–8.43%, indicating that a major gene in this genomic interval may improve seed germination (**Table 3**). Interestingly, 12 associated SNPs were located within the most significant haplotype block, which was almost 920 kb long and contained five haplotypes (**Figures 5A, B**). A haplotype analysis revealed that *qGI/GP/GR-D03-3* had two major haplotypes according to SNP location. Comparatively, Hap1 had a higher GP than Hap1 (**Figures 5C, D**). In total, 46 candidate genes contained in the *qGI/GP/GR-D03-3* region on chromosome D03 were identified. Among them, *Ghir_D03G009280* was annotated as auxin response factor 9 (*ARF9*) in *Arabidopsis* (**Supplementary Table S6**), and its homologs played a crucial role in seed dormancy. The RNA-seq and qRT-PCR assays also showed that *Ghir_D03G009280* had higher expression levels during the seed germination stage, suggesting a positive regulatory effect (**Figure 5E**; **Supplementary Figure S5**).

## Discussion

### The importance of seed vigor for field production

SV is an indispensable indicator of seed quality, which directly affects the rapid and uniform germination of seeds and the robust growth of seedlings and affects the tolerance of plants to abiotic stress in the early stage of seedling growth (Qun et al., 2007; Fujino et al., 2008). In recent years, mechanical DS of cotton has been widely used due to its cost-saving and labor-saving advantages, leading to rapid and uniform seed germination becoming necessary conditions for high yield and mechanization in the cotton industry. However, seeds with low SV make it difficult for mechanical DS to achieve full seeding, which leads to problems such as subsequent filling of the gaps with seedlings and final singling of seedlings (Xie et al., 2014)—for example, Xinjiang Province is the major cotton-growing area in China and experiences serious saline–alkali stress (He et al., 2023). A high SV of cotton varieties will improve seed germination in the field and thus increase the yield. In addition, cotton breeding without plastic film in Xinjiang Province to eliminate “white pollution” has become popular. The germination rate and seedling emergence rate of seeds have higher requirements for cotton without plastic film (CWPF). CWPF needs to quickly establish robust seedlings after seed germination to resist the invasion of diseases, insect pests, adverse environments, and other factors in the field. Importantly, SV is the result of genetic and environmental factors and is thus often difficult to effectively select in conventional breeding (Dai et al., 2022). This study utilized high-throughput sequencing to generate widely distributed SNP markers that cover the whole genome (**Figure 1**), and over 200,000,000 high-quality SNPs were detected in a diverse set of 355 cotton accessions. Combining phenotype data from multiple environments for GWAS analysis can be used to effectively identify genetic loci and candidate genes that improve SV in upland cotton, providing an effective way to improve cotton yield in Xinjiang when using the MAS method.

### Comprehensive analysis of SV-related traits at multiple environments

To ensure the accuracy of the GWAS results, phenotypic identification in multiple environments was conducted with at least three replicates per environment. The three SV-related traits (GI, GP, and GR) were measured for seeds collected from three locations: E1, E2, and E3. Among them, GR and GP did not show an absolute normal distribution, which was also found in previous studies (Dai et al., 2022; Si et al., 2022), indicating a complex genetic basis for these SV-related traits. Through phenotypic correlation analysis, it was found that there were significant positive correlations between the three traits. The GI showed a strong correlation with GR and GP (0.71 and 0.76, respectively) (**Figure 2J**). The highest GI was accompanied by the highest GP and GR, which is consistent with previous findings (Si et al., 2022). Furthermore, according to the measurement results for each trait, the CV of SV-related traits in upland cotton is affected by the environment (**Supplementary Table S2**), resulting in different variations in the seeds of each accession harvested in different planting locations and years—for example, the CV of the GI and GR in E1 showed a larger range of variation than that in E2 and E3. Previous studies have shown that the environment in the planting area has a great influence on the growth and development of seeds (Fenner, 1992). It is speculated that the E2 and E3 (Sanya City, Hainan Province) environments with tropical climates are more suitable environments for seed growth, and the performance of the seeds may be relatively stable. In contrast, the E1 environment (Huanggang City, Hubei Province) has high precipitation and temperature during the seed maturation period, which can affect the success of pollination.

### Candidate genes related to SV

In the past two decades, GWAS has become a powerful and widely used tool for analyzing the genetic mechanisms underlying complex quantitative traits in crops (Tibbs Cortes et al., 2021). At present, most research on SV mainly focuses on the mechanism under stress in upland cotton (Sun et al., 2018; Yuan et al., 2019; Zheng et al., 2021), while genetic analysis of SV-related traits associated with normal seed germination is less common (Si et al., 2022). In this study, a GWAS panel was used to measure three SV-related traits of seeds harvested in three environments. In total, six significant QTLs were stably identified on three different cotton chromosomes (**Table 3**), including 26 SNPs. Numerous studies have reported that several pathways are involved in regulating SV in plants, such as phytohormone signaling (GA, ABA, and auxin), amino acid metabolism, and the reactive oxygen pathway, which play a crucial role in the seed germination process and have a significant effect on the molecular mechanisms related to SV (Reed et al., 2022). It has been reported that high concentrations of ABA promote dormancy and inhibit seed germination, while high concentrations of GA promote seed germination by reversing dormancy, leading to an endogenous balance of the ABA/GA ratio but not the absolute hormone contents (Finch-Savage and Leubner-Metzger, 2006; Chen H. et al., 2020). *Ghir_A09G002650* was annotated on chromosome A09, belonging to the GA-regulated family of proteins and encoding a protein containing the GASA domain, which is most closely related to the known homolog *GASA14* in *Arabidopsis. GASA14* regulates the increase in plant growth through GA induction and DELLA-dependent signal transduction, which could increase resistance to abiotic stress by reducing the accumulation of ROS (Sun et al., 2013). Thus, it is speculated that *Ghir_A09G002650* has the potential to improve the SV of cotton under stress. MYB-type and bHLH-type transcription factors have been reported to be involved in the regulation of seed germination signaling in plants (Penfield et al., 2005; Reyes and Chua, 2007; Kim et al., 2015; Wang X. et al., 2022; Xu et al., 2022). Specifically, *Ghir_D03G006550* is in the *qGI/GR-D03-2* region and is homologous to *MYB52*. It has been previously shown that its shared common targets with *ERF4* regulate the development of the seed coat in *Arabidopsis* (Ding et al., 2021). *Ghir_D03G010510* encoded bHLH-type family proteins in the QTL region of *qGI/GP/GR-D03-4*, sharing 35.52% sequence identity with the PIF8 protein in *Arabidopsis*, which binds to promoter regions of AtPIF6. The expression level of *AtPIF6* during seed development plays a crucial role in establishing primary seed dormancy levels (Peters et al., 2010).

Notably, *Ghir_A09G002730* and *Ghir_D03G009280* were detected in two distinct enriched regions located on chromosome A09 (*qGR-A09-1*) and chromosome D03 (*qGI/GP/GR-D03-3*) (**Figure 3**). Interestingly, *Ghir_A09G002730*, within the strong-LD region at 21.9 kb upstream of rsA09_7962794 and highly expressed during the development of seed germination (**Figures 4A, E**), encodes a PPR superfamily protein in *Arabidopsis*. *SOAR1* belongs to the PPR protein family and acts as a core negative regulator downstream of *ABAR* and upstream of *ABI5*, participating in ABA signaling regulation of seed germination and seedling growth processes (Ma et al., 2020). We also discovered that cotton accessions carrying rsA09_7962794-A with a higher GR had a much higher allele frequency for *Ghir_A09G002730* in YZRR and NIR than in YRR and NSER (**Figures 4B, C**). It is possible that the planting mode of seedling raising and transplanting in YZRR and mechanized planting in the NIR all employed single-seed sowing, which increased the selection frequency of rsA09_7962794-A. In addition, we compared the genetic diversity of the region on chromosome A09 containing *Ghir_A09G002730* in different breeding periods, and it was found that cultivars bred after the 2000s had lower genetic diversity than cultivars from other stages, implying that with the continuous increase in cotton SV during the breeding process, this gene was associated with artificial selection (**Figure 4D**). Therefore, it is reasonable to postulate that *Ghir_A09G002730* is a new candidate gene influencing SV in cotton. *Ghir_D03G009280* caught our attention based on the gene annotation of cotton. This gene encodes an auxin response factor. Recent studies have shown that *ARF16* interacts with *ABI5* and positively regulates the ABA response during seed germination (Mei et al., 2023). Furthermore, *Ghir D03G009280*, tightly linked with haplotype Hap1, showed a significant association with GP (**Figure 5C**), and materials carrying the Hap1 haplotype had longer roots (**Figure 5D**). The RNA-seq analysis showed a high expression level of this gene during seed germination (**Figure 5E**). From the above-mentioned results, we inferred that *Ghir_A09G002730* and *Ghir_D03G009280* were two major candidate genes that may play an important role in cotton SV.

## Conclusions

In the present study, there was a total of 121 predicted candidate genes within six stable QTL regions. Furthermore, *Ghir_A09G002730* and *Ghir_D03G009280* caught our attention based on gene expression (RNA-seq and qRT-PCR), gene annotation, and haplotype analysis, which may play a key role in regulating the germination of cotton seeds. These results will enhance our understanding of the molecular–genetic regulation of SV in cotton.

## Data availability statement

The datasets presented in this study can be found in online repositories. The names of the repository/repositories and accession number(s) can be found below: The resequencing data (PRJNA389777) of the 355 upland cotton germplasms used in this study. For RNA-seq data: The public RNA-seq data (PRJNA248163) including SRR1695160, SRR1695161, and SRR1695162 were downloaded from the NCBI (https://www.ncbi.nlm.nih.gov/bioproject/).

## Author contributions

LL: Conceptualization, Software, Visualization, Writing – original draft, Writing – review & editing. YH: Data curation, Formal Analysis, Investigation, Software, Visualization, Writing – original draft. YW: Methodology, Writing – review & editing. YY: Writing – original draft. RL: Software, Visualization, Writing – review & editing. JW: Visualization, Writing – review & editing. MY: Methodology, Supervision, Writing – review & editing. SZ: Conceptualization, Investigation, Software, Writing – review & editing. FZ: Writing – review & editing. JH: Conceptualization, Writing – review & editing. SY: Conceptualization, Writing – review & editing. ZF: Conceptualization, Investigation, Supervision, Writing – review & editing.

## References

[B1] Andrews S. (2010). “FastQC: a quality control tool for high throughput sequence data,” Babraham Bioinformatics (United Kingdom: Babraham Bioinformatics, Babraham Institute, Cambridge).

[B2] Chen L. Liu L. Lu B. Ma T. Jiang D. Li J.. (2020). Exogenous melatonin promotes seed germination and osmotic regulation under salt stress in cotton (*Gossypium hirsutum* L.). PloS One 15, e0228241. doi: 10.1371/journal.pone.0228241 32004326 PMC6994006

[B3] Chen H. Ruan J. Chu P. Fu W. Liang Z. Li Y.. (2020). AtPER1 enhances primary seed dormancy and reduces seed germination by suppressing the ABA catabolism and GA biosynthesis in Arabidopsis seeds. Plant J. 101, 310–323. doi: 10.1111/tpj.14542 31536657

[B4] Chen Z. J. Scheffler B. E. Dennis E. Triplett B. A. Zhang T. Guo W.. (2007). Toward sequencing cotton (*Gossypium*) genomes. Plant Physiol. 145, 1303–1310. doi: 10.1104/pp.107.107672 18056866 PMC2151711

[B5] Chen S. Zhou Y. Chen Y. Gu J. (2018). fastp: an ultra-fast all-in-one FASTQ preprocessor. Bioinformatics 34, i884–i890. doi: 10.1093/bioinformatics/bty560 30423086 PMC6129281

[B6] Dai L. Lu X. Shen L. Guo L. Zhang G. Gao Z.. (2022). Genome-wide association study reveals novel QTLs and candidate genes for seed vigor in rice. Front. Plant Sci. 13. doi: 10.3389/fpls.2022.1005203 PMC9645239 36388599

[B7] Danecek P. Auton A. Abecasis G. Albers C. A. Banks E. Depristo M. A.. (2011). The variant call format and VCFtools. Bioinformatics 27, 2156–2158. doi: 10.1093/bioinformatics/btr330 21653522 PMC3137218

[B8] Danecek P. Bonfield J. K. Liddle J. Marshall J. Ohan V. Pollard M. O.. (2021). Twelve years of SAMtools and BCFtools. Gigascience 10, giab008. doi: 10.1093/gigascience/giab008 33590861 PMC7931819

[B9] Dimaano N. G. B. Ali J. Mahender A. Sta. Cruz P. C. Baltazar A. M. Diaz M. G. Q.. (2020). Identification of quantitative trait loci governing early germination and seedling vigor traits related to weed competitive ability in rice. Euphytica 216, 159. doi: 10.1007/s10681-020-02694-8 33029032 PMC7510932

[B10] Ding A. Tang X. Yang D. Wang M. Ren A. Xu Z.. (2021). ERF4 and MYB52 transcription factors play antagonistic roles in regulating homogalacturonan de-methylesterification in Arabidopsis seed coat mucilage. Plant Cell 33, 381–403. doi: 10.1093/plcell/koaa031 33709105 PMC8136884

[B11] Feng Z. Li L. Tang M. Liu Q. Ji Z. Sun D.. (2022). Detection of stable elite haplotypes and potential candidate genes of boll weight across multiple environments via GWAS in upland cotton. Front. Plant Sci. 13. doi: 10.3389/fpls.2022.929168 PMC9234699 35769298

[B12] Fenner M. (1992). Environmental influences on seed size and composition. Hortic. Rev. 13, 183–213. doi: 10.1002/9780470650509.ch5

[B13] Finch-Savage W. E. Leubner-Metzger G. (2006). Seed dormancy and the control of germination. New Phytol. 171, 501–523. doi: 10.1111/j.1469-8137.2006.01787.x 16866955

[B14] Fujino K. Sekiguchi H. Matsuda Y. Sugimoto K. Ono K. Yano M. (2008). Molecular identification of a major quantitative trait locus, qLTG3–1, controlling low-temperature germinability in rice. Proc. Natl. Acad. Sci. U.S.A. 105, 12623–12628. doi: 10.1073/pnas.0805303105 18719107 PMC2527961

[B15] Fujino K. Sekiguchi H. Sato T. Kiuchi H. Nonoue Y. Takeuchi Y.. (2004). Mapping of quantitative trait loci controlling low-temperature germinability in rice (Oryza sativa L.). Theor. Appl. Genet. 108, 794–799. doi: 10.1007/s00122-003-1509-4 14624339

[B16] Gu Q. Ke H. Liu C. Lv X. Sun Z. Liu Z.. (2021). A stable QTL qSalt-A04-1 contributes to salt tolerance in the cotton seed germination stage. Theor. Appl. Genet. 134, 2399–2410. doi: 10.1007/s00122-021-03831-0 33928409

[B17] Guo A. Su Y. Nie H. Li B. Ma X. Hua J. (2022). Identification of candidate genes involved in salt stress response at germination and seedling stages by QTL mapping in upland cotton. G3 (Bethesda) 12, jkac099. doi: 10.1093/g3journal/jkac099 35471243 PMC9157077

[B18] He Y. Cheng J. He Y. Yang B. Cheng Y. Yang C.. (2019a). Influence of isopropylmalate synthase Os IPMS 1 on seed vigour associated with amino acid and energy metabolism in rice. Plant Biotechnol. J. 17, 322–337. doi: 10.1111/pbi.12979 29947463 PMC6335077

[B19] He P. Li J. Yu S. E. Ma T. Ding J. Zhang F.. (2023). Soil moisture regulation under mulched drip irrigation influences the soil salt distribution and growth of cotton in Southern Xinjiang, China. Plants 12, 791. doi: 10.3390/plants12040791 36840139 PMC9964176

[B20] He Y. Yang B. He Y. Zhan C. Cheng Y. Zhang J.. (2019b). A quantitative trait locus, qSE 3, promotes seed germination and seedling establishment under salinity stress in rice. Plant J. 97, 1089–1104. doi: 10.1111/tpj.14181 30537381 PMC6850641

[B21] Iglesias-Fernandez R. Matilla A. (2009). After-ripening alters the gene expression pattern of oxidases involved in the ethylene and gibberellin pathways during early imbibition of Sisymbrium officinale L. seeds. J. Exp. Bot. 60, 1645–1661. doi: 10.1093/jxb/erp029 19246594 PMC2671615

[B22] Ishibashi Y. Yamamoto K. Tawaratsumida T. Yuasa T. Iwaya-Inoue M. (2008). Hydrogen peroxide scavenging regulates germination ability during wheat (Triticum aestivum L.) seed maturation. Plant Signal Behav. 3, 183–188. doi: 10.4161/psb.3.3.5540 19513213 PMC2634112

[B23] Jiang S. Yang C. Xu Q. Wang L. Yang X. Song X.. (2020). Genetic dissection of germinability under low temperature by building a resequencing linkage map in japonica rice. Int. J. Mol. Sci. 21, 1284. doi: 10.3390/ijms21041284 32074988 PMC7072905

[B24] Johnson M. Zaretskaya I. Raytselis Y. Merezhuk Y. Mcginnis S. Madden T. L. (2008). NCBI BLAST: a better web interface. Nucleic Acids Res. 36, W5–W9. doi: 10.1093/nar/gkn201 18440982 PMC2447716

[B25] Kim T.-H. Böhmer M. Hu H. Nishimura N. Schroeder J. I. (2010). Guard cell signal transduction network: advances in understanding abscisic acid, CO2, and Ca2+ signaling. Annu. Rev. Plant Biol. 61, 561–591. doi: 10.1146/annurev-arplant-042809-112226 20192751 PMC3056615

[B26] Kim J. H. Hyun W. Y. Nguyen H. N. Jeong C. Y. Xiong L. Hong S. W.. (2015). AtMyb7, a subgroup 4 R2R3 Myb, negatively regulates ABA-induced inhibition of seed germination by blocking the expression of the bZIP transcription factor ABI 5. Plant Cell Environ. 38, 559–571. doi: 10.1111/pce.12415 25053018

[B27] Kolde R. (2012). Pheatmap: pretty heatmaps. R Package version 1, 726.

[B28] Leymarie J. Vitkauskaité G. Hoang H. H. Gendreau E. Chazoule V. Meimoun P.. (2012). Role of reactive oxygen species in the regulation of Arabidopsis seed dormancy. Plant Cell Physiol. 53, 96–106. doi: 10.1093/pcp/pcr129 21937678

[B29] Li H. (2013). Aligning sequence reads, clone sequences and assembly contigs with BWA-MEM. arXiv preprint. arXiv, 1303.3997. doi: 10.48550/arXiv.1303.3997

[B30] Li W. Niu Y. Zheng Y. Wang Z. (2022). Advances in the understanding of reactive oxygen species-dependent regulation on seed dormancy, germination, and deterioration in crops. Front. Plant Sci. 13. doi: 10.3389/fpls.2022.826809 PMC8905223 35283906

[B31] Li W. Yang B. Xu J. Peng L. Sun S. Huang Z.. (2021). A genome-wide association study reveals that the 2-oxoglutarate/malate translocator mediates seed vigor in rice. Plant J. 108, 478–491. doi: 10.1111/tpj.15455 34376020

[B32] Li L. Zhang C. Huang J. Liu Q. Wei H. Wang H.. (2021). Genomic analyses reveal the genetic basis of early maturity and identification of loci and candidate genes in upland cotton (Gossypium hirsutum L.). Plant Biotechnol. J. 19, 109–123. doi: 10.1111/pbi.13446 32652678 PMC7769233

[B33] Li L. Zhao S. Su J. Fan S. Pang C. Wei H.. (2017). High-density genetic linkage map construction by F2 populations and QTL analysis of early-maturity traits in upland cotton (Gossypium hirsutum L.). PloS One 12, e0182918. doi: 10.1371/journal.pone.0182918 28809947 PMC5557542

[B34] Liu H. Hussain S. Zheng M. Peng S. Huang J. Cui K.. (2015). Dry direct-seeded rice as an alternative to transplanted-flooded rice in Central China. Agron. Sustain. Dev. 35, 285–294. doi: 10.1007/s13593-014-0239-0

[B35] Ma Y. Zhang S. Bi C. Mei C. Jiang S.-C. Wang X.-F.. (2020). Arabidopsis exoribonuclease USB1 interacts with the PPR-domain protein SOAR1 to negatively regulate abscisic acid signaling. J. Exp. Bot. 71, 5837–5851. doi: 10.1093/jxb/eraa315 32969475 PMC7541913

[B36] Mckenna A. Hanna M. Banks E. Sivachenko A. Cibulskis K. Kernytsky A.. (2010). The Genome Analysis Toolkit: a MapReduce framework for analyzing next-generation DNA sequencing data. Genome Res. 20, 1297–1303. doi: 10.1101/gr.107524.110 20644199 PMC2928508

[B37] Mei S. Zhang M. Ye J. Du J. Jiang Y. Hu Y. (2023). Auxin contributes to jasmonate-mediated regulation of abscisic acid signaling during seed germination in Arabidopsis. Plant Cell 35, 1110–1133. doi: 10.1093/plcell/koac362 36516412 PMC10015168

[B38] Penfield S. Josse E.-M. Kannangara R. Gilday A. D. Halliday K. J. Graham I. A. (2005). Cold and light control seed germination through the bHLH transcription factor SPATULA. Curr. Biol. 15, 1998–2006. doi: 10.1016/j.cub.2005.11.010 16303558

[B39] Peng L. Sun S. Yang B. Zhao J. Li W. Huang Z.. (2022). Genome-wide association study reveals that the cupin domain protein OsCDP3. 10 regulates seed vigour in rice. Plant Biotechnol. J. 20, 485–498. doi: 10.1111/pbi.13731 34665915 PMC8882794

[B40] Peters S. Egert A. Stieger B. Keller F. (2010). Functional identification of Arabidopsis ATSIP2 (At3g57520) as an alkaline α-galactosidase with a substrate specificity for raffinose and an apparent sink-specific expression pattern. Plant Cell Physiol. 51, 1815–1819. doi: 10.1093/pcp/pcq127 20739305

[B41] Qun S. Wang J.-H. Sun B.-Q. (2007). Advances on seed vigor physiological and genetic mechanisms. Agric. Sci. China 6, 1060–1066. doi: 10.1016/S1671-2927(07)60147-3

[B42] Reed R. C. Bradford K. J. Khanday I. (2022). Seed germination and vigor: ensuring crop sustainability in a changing climate. Heredity 128, 450–459. doi: 10.1038/s41437-022-00497-2 35013549 PMC9177656

[B43] Reyes J. L. Chua N. H. (2007). ABA induction of miR159 controls transcript levels of two MYB factors during Arabidopsis seed germination. Plant J. 49, 592–606. doi: 10.1111/j.1365-313X.2006.02980.x 17217461

[B44] Ryu H. Cho Y.-G. (2015). Plant hormones in salt stress tolerance. J. Plant Biol. 58, 147–155. doi: 10.1007/s12374-015-0103-z

[B45] Sawan Z. M. (2016). Cottonseed yield and its quality as affected by mineral nutrients and plant growth retardants. Cogent Biol. 2, 1245938. doi: 10.1080/23312025.2016.1245938

[B46] Shi H. Guan W. Shi Y. Wang S. Fan H. Yang J.. (2020). QTL mapping and candidate gene analysis of seed vigor-related traits during artificial aging in wheat (Triticum aestivum). Sci. Rep. 10, 1–13. doi: 10.1038/s41598-020-75778-z 33328518 PMC7745025

[B47] Shikha K. Shahi J. Vinayan M. Zaidi P. Singh A. Sinha B. (2021). Genome-wide association mapping in maize: status and prospects. 3 Biotech. 11, 244. doi: 10.1007/s13205-021-02799-4 PMC8085158 33968587

[B48] Shin J.-H. Blay S. Mcneney B. Graham J. (2006). LDheatmap: an R function for graphical display of pairwise linkage disequilibria between single nucleotide polymorphisms. J. Stat. Softw. 16, 1–9. doi: 10.18637/jss.v016.c03

[B49] Si A. Sun Z. Li Z. Chen B. Gu Q. Zhang Y.. (2022). A genome wide association study revealed key single nucleotide polymorphisms/genes associated with seed germination in Gossypium hirsutum L. Front. Plant Sci. 13, 844946. doi: 10.3389/fpls.2022.844946 35371175 PMC8967292

[B50] Su J. Fan S. Li L. Wei H. Wang C. Wang H.. (2016a). Detection of favorable QTL alleles and candidate genes for lint percentage by GWAS in Chinese upland cotton. Front. Plant Sci. 7. doi: 10.3389/fpls.2022.844946 PMC5073211 27818672

[B51] Su J. Li L. Pang C. Wei H. Wang C. Song M.. (2016b). Two genomic regions associated with fiber quality traits in Chinese upland cotton under apparent breeding selection. Sci. Rep. 6, 1–14. doi: 10.1038/srep38496 27924947 PMC5141495

[B52] Su J. Li L. Zhang C. Wang C. Gu L. Wang H.. (2018). Genome-wide association study identified genetic variations and candidate genes for plant architecture component traits in Chinese upland cotton. Theor. Appl. Genet. 131, 1299–1314. doi: 10.1007/s00122-018-3079-5 29497767

[B53] Sun Z. Li H. Zhang Y. Li Z. Ke H. Wu L.. (2018). Identification of SNPs and candidate genes associated with salt tolerance at the seedling stage in cotton (*Gossypium hirsutum* L.). Front. Plant Sci. 9. doi: 10.3389/fpls.2018.01011 PMC6050395 30050555

[B54] Sun S. Wang H. Yu H. Zhong C. Zhang X. Peng J.. (2013). GASA14 regulates leaf expansion and abiotic stress resistance by modulating reactive oxygen species accumulation. J. Exp. Bot. 64, 1637–1647. doi: 10.1093/jxb/ert021 23378382

[B55] Tibbs Cortes L. Zhang Z. Yu J. (2021). Status and prospects of genome-wide association studies in plants. Plant Genome 14, e20077. doi: 10.1002/tpg2.20077 33442955 PMC12806871

[B56] Veisi S. Sabouri A. Abedi A. (2022). Meta-analysis of QTLs and candidate genes associated with seed germination in rice (*Oryza sativa* L.). Physiol. Mol. Biol. Plants 28, 1587–1605. doi: 10.1007/s12298-022-01232-1 36389095 PMC9530108

[B57] Vogt F. Shirsekar G. Weigel D. (2022). vcf2gwas: Python API for comprehensive GWAS analysis using GEMMA. Bioinformatics 38, 839–840. doi: 10.1093/bioinformatics/btab710 34636840 PMC8756188

[B58] Wang L. Li J. Yang F. Dai D. Li X. Sheng Y. (2022). A preliminary mapping of QTL qsg5. 1 controlling seed germination in melon (Cucumis melo L.). Front. Plant Sci. 13. doi: 10.3389/fpls.2022.925081 PMC9421157 36046593

[B59] Wang M. Tu L. Yuan D. Zhu D. Shen C. Li J.. (2019). Reference genome sequences of two cultivated allotetraploid cottons, *Gossypium hirsutum* and *Gossypium barbadense* . Nat. Genet. 51, 224–229. doi: 10.1038/s41588-018-0282-x 30510239

[B60] Wang X. Wu R. Shen T. Li Z. Li C. Wu B.. (2022). An R2R3-MYB transcription factor OsMYBAS1 promotes seed germination under different sowing depths in transgenic rice. Plants 11, 139. doi: 10.3390/plants11010139 35009142 PMC8747419

[B61] Wang X. Zou B. Shao Q. Cui Y. Lu S. Zhang Y.. (2018). Natural variation reveals that OsSAP16 controls low-temperature germination in rice. J. Exp. Bot. 69, 413–421. doi: 10.1093/jxb/erx413 29237030 PMC5853544

[B62] Wickham H. (2011). ggplot2. Wiley interdisciplinary reviews: computational statistics Wiley interdisciplinary reviews: computationalstatistics 3, 180–185. doi: 10.1002/wics.147

[B63] Xie L. Tan Z. Zhou Y. Xu R. Feng L. Xing Y.. (2014). Identification and fine mapping of quantitative trait loci for seed vigor in germination and seedling establishment in rice. J. Integr. Plant Biol. 56, 749–759. doi: 10.1111/jipb.12190 24571491

[B64] Xu F. Tang J. Wang S. Cheng X. Wang H. Ou S.. (2022). Antagonistic control of seed dormancy in rice by two bHLH transcription factors. Nat. Genet. 54, 1972–1982. doi: 10.1038/s41588-022-01240-7 36471073

[B65] Yamaguchi S. (2008). Gibberellin metabolism and its regulation. Annu. Rev. Plant Biol. 59, 225–251. doi: 10.1146/annurev.arplant.59.032607.092804 18173378

[B66] Yamauchi Y. Ogawa M. Kuwahara A. Hanada A. Kamiya Y. Yamaguchi S. (2004). Activation of gibberellin biosynthesis and response pathways by low temperature during imbibition of Arabidopsis thaliana seeds. Plant Cell 16, 367–378. doi: 10.1105/tpc.018143 14729916 PMC341910

[B67] Ye N. Zhu G. Liu Y. Zhang A. Li Y. Liu R.. (2012). Ascorbic acid and reactive oxygen species are involved in the inhibition of seed germination by abscisic acid in rice seeds. J. Exp. Bot. 63, 1809–1822. doi: 10.1093/jxb/err336 22200664 PMC3295380

[B68] Yuan Y. Xing H. Zeng W. Xu J. Mao L. Wang L.. (2019). Genome-wide association and differential expression analysis of salt tolerance in *Gossypium hirsutum* L at the germination stage. BMC Plant Biol. 19, 1–19. doi: 10.1186/s12870-019-1989-2 31510912 PMC6737726

[B69] Zhang C. Li L. Liu Q. Gu L. Huang J. Wei H.. (2019). Identification of loci and candidate genes responsible for fiber length in upland cotton (*Gossypium hirsutum* L.) via association mapping and linkage analyses. Front. Plant Sci. 10. doi: 10.3389/fpls.2019.00053 PMC6370998 30804954

[B70] Zheng J. Zhang Z. Gong Z. Liang Y. Sang Z. Xu Y.. (2021). Genome-wide association analysis of salt-tolerant traits in terrestrial cotton at seedling stage. Plants 11, 97. doi: 10.3390/plants11010097 35009100 PMC8747425

[B71] Zhou X. Stephens M. (2012). Genome-wide efficient mixed-model analysis for association studies. Nat. Genet. 44, 821–824. doi: 10.1038/ng.2310 22706312 PMC3386377

[B72] Zhu C. Gore M. Buckler E. S. Yu J. (2008). Status and prospects of association mapping in plants. Plant Genome 1. doi: 10.3835/plantgenome2008.02.0089

