# Supplementary material for: Identification of novel candidate loci and genes for seed vigor-related traits in upland cotton (Gossypium hirsutum L.) via GWAS
Source: Front Plant Sci. 2023 Sep 1;14:1254365. doi: 10.3389/fpls.2023.1254365 (PMC10503134; doi:10.3389/fpls.2023.1254365)
Supplement: Supplementary file 2 [file Image_1.pdf]

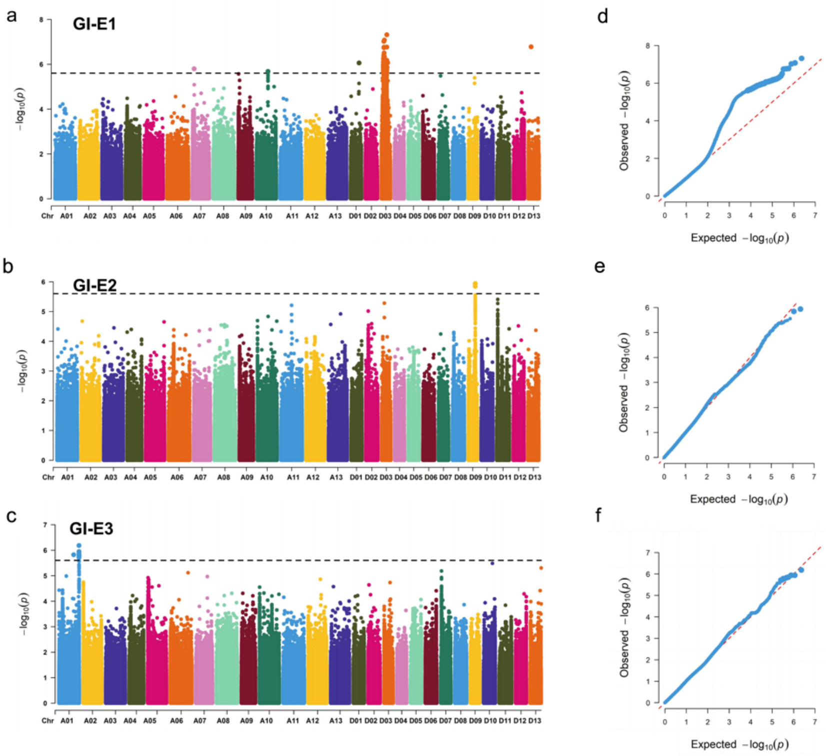

Fig. S1: Manhattan and QQ plots for GI in each separate environment

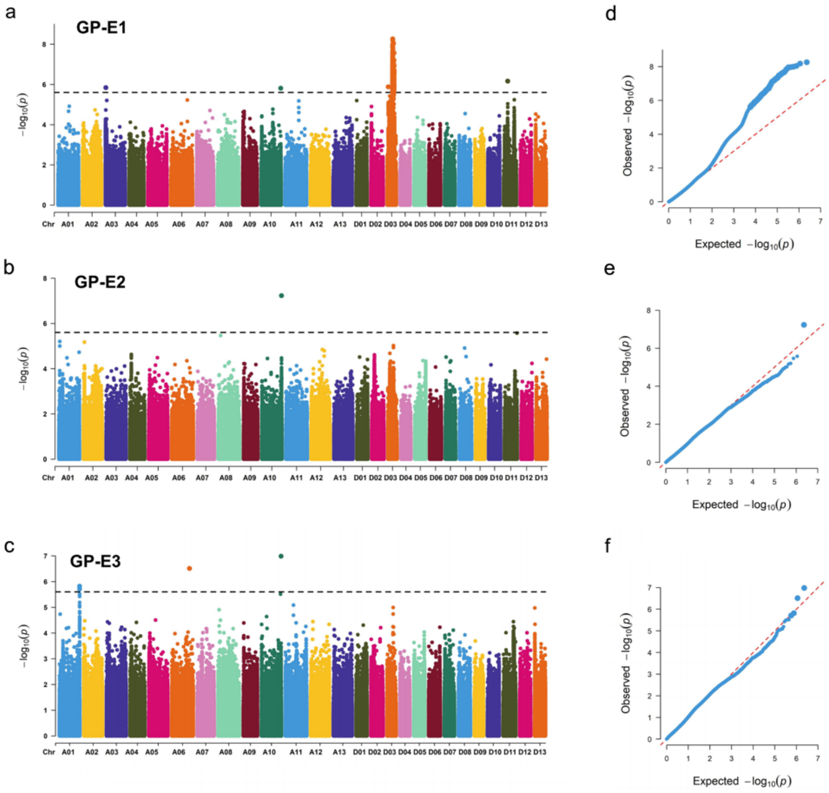

Fig. S2: Manhattan and QQ plots for GP in each separate environment

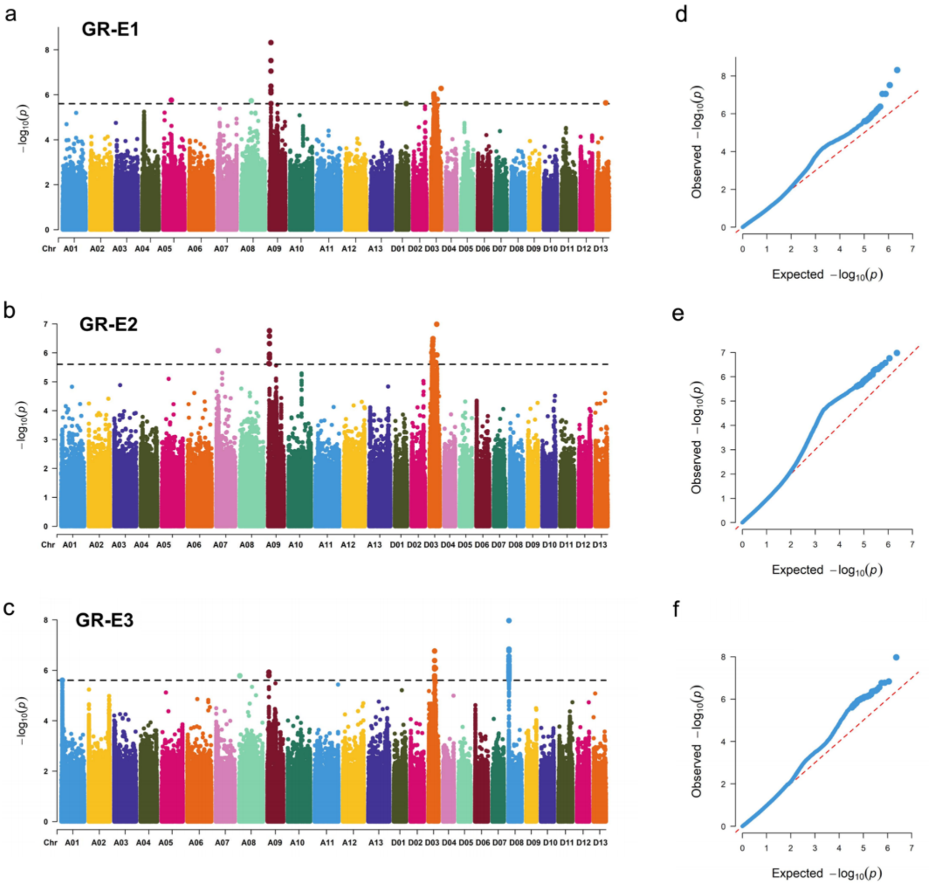

Fig. S3: Manhattan and QQ plots for GR in each separate environment

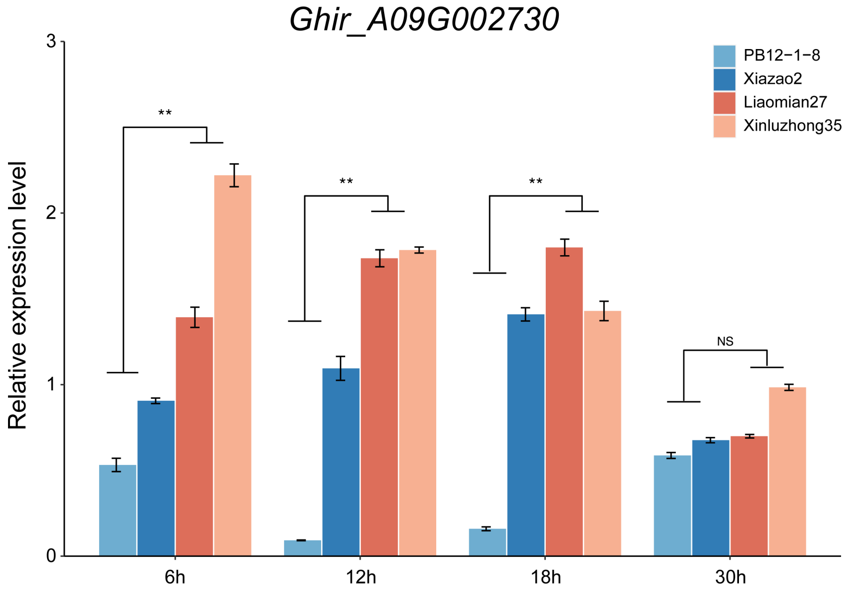

Fig. S4: Expression level analysis of *Ghir_ A09G002730* between “Liaomian27’, ‘Xinluzhong35’, ‘PB12-1-8’, and ‘Xiazao2’ during seed germination stage (6, 12, 18 and 30 h) by qRT–PCR (** $P < 0.01$, * $P < 0.05$).

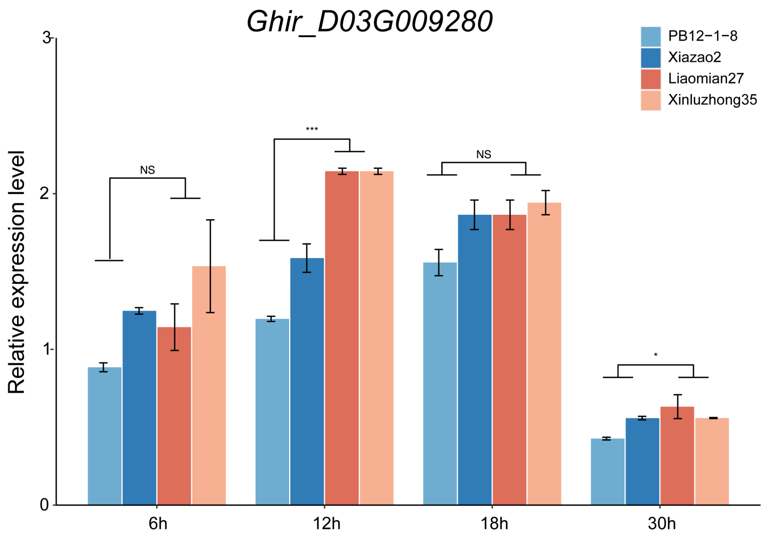

Fig. S5: Expression level analysis of *Ghir_ D03G009280* between ''Liaomian27', 'Xinluzhong35', 'PB12-1-8', and 'Xiazao2' during seed germination stage (6, 12, 18 and 30 h) by qRT–PCR (** P < 0.01, * P < 0.05).
